# Prognostic Factors for All-Cause Mortality in Thai Patients with Fragility Fracture of Hip: Comorbidities and Laboratory Evaluations

**DOI:** 10.3390/medicina56060311

**Published:** 2020-06-24

**Authors:** Pichitchai Atthakomol, Worapaka Manosroi, Phichayut Phinyo, Tanyong Pipanmekaporn, Tanawat Vaseenon, Sattaya Rojanasthien

**Affiliations:** 1 Department of Orthopaedics, Faculty of Medicine, Chiang Mai University, Chiang Mai 50200, Thailand; p.atthakomol@gmail.com (P.A.); tvaseenon@gmail.com (T.V.); srojanas@gmail.com (S.R.); 2 Musculoskeletal Science and Translational Research Center, Chiang Mai University, Chiang Mai 50200, Thailand; 3 Division of Endocrinology, Department of Internal Medicine, Faculty of Medicine, Chiang Mai University, Chiang Mai 50200, Thailand; worapaka.m@gmail.com; 4 Department of Family Medicine, Faculty of Medicine, Chiang Mai University, Chiang Mai 50200, Thailand; phichayutphinyo@gmail.com; 5 Center for Clinical Epidemiology and Clinical Statistics, Faculty of Medicine, Chiang Mai University, Chiang Mai 50200, Thailand; 6 Department of Anesthesiology, Faculty of Medicine, Chiang Mai University, Chiang Mai 50200, Thailand

**Keywords:** prognostic factors, mortality, Thai, hip fracture, comorbidities, laboratory evaluations

## Abstract

*Background and Objectives*: Although the types of comorbidities and laboratory evaluations are major factors associated with mortality after hip fractures, there have been no studies of the association of these factors and mortality in Thai hip-fracture patients. This study aimed to identify prognostic factors associated with mortality after a hip fracture in the Thai population, including types of comorbidities, treatment-related factors, and laboratory evaluations. *Materials and Methods*: This five-year retrospective study was conducted in a tertiary care hospital in Thailand. A total of 775 Thai patients who had been admitted with a hip fracture resulting from a simple fall were identified using the International Classification of Disease 10 codes, and a review of their medical charts was conducted. Associations between general factors, comorbidities, laboratory evaluations, treatment factors including type of treatment, and time to death were analyzed using the Cox proportional hazard regression and the hazard ratio (HR). *Results:* The overall mortality rate of hip fracture patients was 13.94%. Independent prognostic factors found to be significantly associated with mortality were nonoperative treatment (HR = 3.29, *p* < 0.001), admission glomerular filtration rate (GFR) < 30 mL/min/1.73 m^2^ (HR = 3.40, *p* < 0.001), admission hemoglobin concentration <10 g/dL. (HR = 2.31, *p* < 0.001), chronic obstructive pulmonary disorder (HR = 2.63, *p* < 0.001), dementia or Alzheimer’s disease (HR = 4.06, *p* < 0.001), and active malignancy (HR = 6.80, *p* < 0.001). *Conclusion:* The types of comorbidities and laboratory evaluation findings associated with mortality in Thai patients with hip fractures include chronic obstructive pulmonary disorder, dementia or Alzheimer’s disease, active malignancy, admission GFR < 30 mL/min/1.73 m^2^, and admission hemoglobin concentration <10 g/dL. The risks of mortality for Thai hip-fracture patients with these comorbidities or laboratory evaluation findings were 2.5, 4, 7, 3.5, and 2.5 times higher, respectively, than patients without those factors.

## 1. Introduction

Worldwide, the expected number of hip fractures in both men and women is increasing exponentially as the size of the elderly population increases [1,2,3]. In patients 50 years old or older, half have had a hip fracture resulting from a simple fall. The incidence increases to 80% for those 75 years old or older [4,5]. The estimated number of hip fractures has been predicted to be 2.6 million by 2025 and to be 4.5 million by 2050 [6].

Short-term and long-term mortality rates of hip fracture patients are common outcome measurements used as quality indicators to evaluate hip fracture care [7,8,9]. Reported one-year mortality incidence varies among different nationalities and other individual factors, ranging from 18.56% to 31% [10,11,12,13,14,15]. Identifying potentially modifiable prognostic factors associated with increased risk of mortality may help guide physicians, patients, and their families to taking appropriate actions to reduce the risk of mortality for individual patients.

Many factors, including age, gender, physical status, institutionalization, comorbidities, findings of laboratory evaluations, method of treatment, and time to surgery have been reported to be associated with in-hospital, 30-day, and 1-year mortality after a hip fracture in diverse populations and countries [3,16,17,18,19,20,21,22,23,24,25,26,27,28,29,30,31,32,33]. In Thailand, data from Chiang Mai University show factors correlated with mortality after hip fracture in the periods 1997 through 1998, 1998 through 2003, and 2006 through 2007 including male gender, greater age, nonoperative treatment, surgical treatment delayed more than a week, chronic illnesses, poor pre-fracture walking ability, and absence of medical treatment for osteoporosis [34,35,36].

Specific types of comorbidities and laboratory evaluation findings have been identified as major factors associated with mortality after a hip fracture [3,16,17,22,23,24,25,26,27,28,29,37,38]. Previous studies of Thai patients, however, have not included these factors in their evaluations of mortality in Thai hip fracture patients [34,35,36]. Additionally, many new factors related to mortality in hip fracture patients have been described in the past decade [24,25,26,27,29,38], suggesting the need for a reevaluation of prognostic factors for all-cause mortality after hip fracture.

The objectives of this study were to evaluate prognostic factors for mortality after hip fracture in the Thai population using the latest data and focusing on types of comorbidities and results of laboratory evaluations and to identify the effect sizes of these factors. 

## 2. Materials and Methods

### 2.1. Study Design

This study was conducted at Academic University Hospital after approval by the institutional ethics committee (ethical number: ORT−256206513; date of approval: 23 August 2019) and followed the Transparent Reporting of a multivariable Prediction model for individual prognosis Or Diagnosis (TRIPOD) Statement [39]. The patient data were obtained from the hospital medical records. International Classification of Disease 10 (ICD-10) codes were used to identify patients who had either a fractured neck of femur (ICD-10 = S7200) or an intertrochanteric fracture (ICD-10 = S7210) and who were admitted to the hospital between January 2014 and December 2018. A member of the hospital’s orthopaedic staff and five senior medical students manually performed the retrospective chart reviews. Any inconclusive data was discussed with three orthopaedic staff members to arrive at a consensus. Inclusion criteria were Thai patients, age at least 50 years, and a hip fracture injury resulting from a simple fall. The exclusion criteria were a bilateral hip fracture, a previous hip fracture, and fractures in more than one area. Any suspected pathological fractures or fractures resulting from a high energy mechanism such as a traffic accident were excluded from the study. Also excluded were patients without a government ID card number issued by the Civil Registration Office as the mortality status of those patients could not be ascertained.

Data, including general demographics, types of comorbidities, type of fracture, laboratory evaluation results, and factors related to treatment were manually gathered from retrospective chart reviews. In cases where the data could not be obtained from medical charts, telephone contact with the patient or their family was made to obtain the information.

All-cause mortality information was obtained from the Thailand Civil Registration Office. In-hospital mortality was counted as all-cause mortality. The definitions of potential prognostic factors are demonstrated in the Supplemental Material.

### 2.2. Statistical Analysis

Categorical variables are reported as frequencies and percentages. Normally distributed continuous variables are shown as means and standard deviations (SD). Non-normally distributed continuous variables are shown as median and interquartile range (IQR). Associations between mortality and categorical variables were calculated using the Fisher’s exact test. Associations with normally distributed continuous variables were evaluated using Student’s t-test. The Mann–Whitney U test was used to analyse the relationship between non-normally distributed variables.

The proportional hazards assumption was tested using Schoenfeld residuals after fitting a model with stcox (estat phtest) for all potential variables.

In univariable analysis, Cox proportional hazard regression was employed to assess the hazard ratio (HR) with a 95% confidence interval (CI) between time to death and potential predictors. Multiple imputation was applied if a potential predictor had >5% missing data [40]. After that, the collinearity of each potential predictor was examined. Predictors which had a variant inflation factor (VIF) value >2 were excluded from the multivariable analysis.

Multivariable analysis to identify independent predictors was conducted using backward elimination to minimize the number of predictors and to preserve the discrimination power of the residual predictors. The discrimination power of the predictors was evaluated using Harrell’s C concordance statistic (C-statistic).

According to the rule of thumb for logistic and Cox models, potential factors should have a minimum of 10 events per predictor variable (EPV) for an adequate sample size [41,42,43,44]. To evaluate the degree of overfitting, bootstrap resampling with 500 replicates was applied to assess the C-statistic optimism. The calibration estimation was demonstrated by the calibration plot.

Statistical analysis was performed using the STATA program (Stata/MP 15.1 for Mac, Copyright 1985-2017, StataCorp LLC). A *p*-value of ˂0.05 was considered statistically significant.

## 3. Results

Of the 1004 patients who were admitted with a hip fracture during the period of this study, 775 (77%) met the inclusion criteria. The mortality rate in that cohort was 13.94% (108 of 775 patients). Most of the patients were female (561 or 72.39%). The average age was 79.09 ± 9.55 years. Most had an intertrochanteric fracture (488 or 62.97%). The median length of stay was 13 ± 9 days. The median follow-up duration was 914 ± 1173 days. Demographic data are shown in Table 1.

All potential predictors except body mass index (BMI) at admission ≥25 kg/meter^2^ followed the proportional hazards assumption.

Univariable analysis of each potential predictor was done using Cox regression analysis (Table 2). The dependent predictors for mortality in Thai patients who had a hip fracture were age ≥ 85 years at admission (HR = 1.83, 95%CI *=* 1.25–2.69, *p* = 0.002), active malignancy (HR = 6.82, 95%CI *=* 3.73–12.47, *p* < 0.001), dementia or Alzheimer’s disease (HR = 2.63, 95%CI *=* 1.47–4.70 *p* = 0.001), chronic obstructive pulmonary disorder (HR = 2.26, 95%CI *=* 1.36–3.75, *p* = 0.002), ASA score > 2 (HR = 3.27, 95%CI *=* 2.07–5.17, *p* < 0.001), admission hemoglobin concentration <10 g/dL. (HR = 2.38, 95%CI *=* 1.63–3.47, *p* < 0.001), admission GFR < 30 mL/min/1.73 m^2^ (HR = 3.10, 95%CI *=* 2.07–4.62, *p* < 0.001), and nonoperative treatment (HR = 3.53, 95%CI *=* 2.30–5.40, *p* < 0.001). Missing data were found in BMI at admission (0.77%), pre-fracture walking ability by oneself (21.81%), living with family (0.13%), and admission serum albumin level (80.52%). Multiple imputations were applied in pre-fracture walking ability by oneself.

To identify independent predictors of mortality after a hip fracture, multivariable analysis of potential predictors was conducted using Cox regression analysis with backward elimination. All potential predictors had a variant inflation factor (VIF) value < 2.

**Figure 1:**
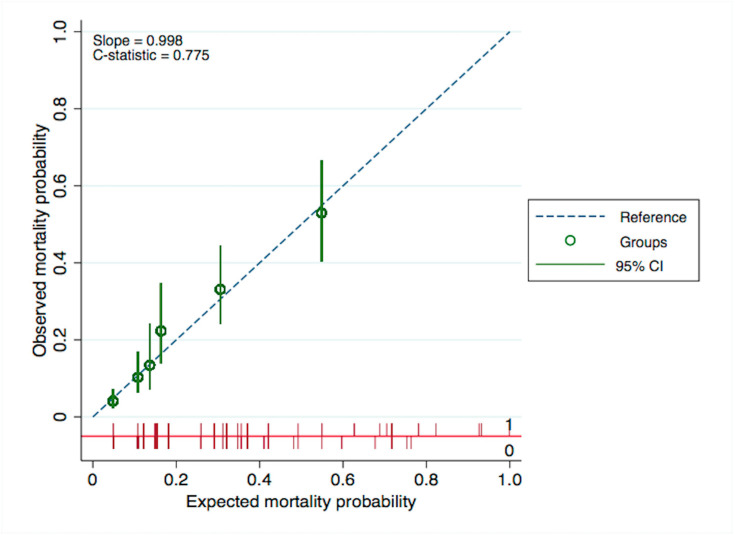
Calibration plot between observed and expected mortality probabilities. CI = Confidence Interval.

The residual independent predictors were nonoperative treatment (HR = 3.29, 95%CI = 2.13–5.08, *p* < 0.001), admission GFR < 30 mL/min/1.73 m^2^ (HR = 3.40, 95%CI = 2.21-5.21, *p* < 0.001), admission hemoglobin concentration <10 g/dL. (HR = 2.31, 95%CI = 1.57–3.40, *p* < 0.001), chronic obstructive pulmonary disorder (HR = 2.63, 95%CI = 1.56–4.42, *p* < 0.001), dementia or Alzheimer’s disease (HR = 4.06, 95%CI = 2.22–7.41, *p* < 0.001), and active malignancy (HR = 6.80, 95%CI = 3.68–12.59, *p* < 0.001) (Table 3). The C-index of these factors was 0.78. The C-statistic optimism was 0.001 (min −0.030, max 0.035) indicated minimal degree of overfitting. The calibration plot showed that the 95%CI of each mortality probability group covered the reference line, suggesting good calibration performance (Figure 1).

## 4. Discussions

Nonoperative treatment, admission GFR < 30 mL/min/1.73 m^2^, admission hemoglobin concentration <10 g/dL, chronic obstructive pulmonary disorder, dementia or Alzheimer’s disease, and active malignancy were significant independent predictors of mortality in Thai patients who had a hip fracture.

In the selection of potential factors, the study adhered to the point before the patient received treatment. Consequently, factors which occurred after this point, e.g., postoperative complications, length of stay, and hospital readmission, were excluded. We did, however, include patients who received conservative treatment. The fact that the prognostic factors identified in our study could apply to all patients receiving various types of treatment provides a benefit in terms of generalizability.

General factor analysis found that patients who were older than 85 years had twice the risk of mortality compared to younger patients. It is clear that advanced age increases the mortality rate, a finding supported by a number of previous studies [3,16,18,19,22,24,25,34,36,38]. However, the effect of advanced age as a prognostic factor for mortality was less than that of other factors, so that factor was finally eliminated as a residual predictor (Table 3). In contrast to the results of earlier studies, living in an institution, gender, and pre-fracture walking ability were found not to be significant prognostic factors for mortality in our cohort [3,16,17,18,19,22,34,36,37]. One reason for this difference could be the strong Thai tradition of respecting and taking care of elder members of the family, e.g., most of the elderly patients were living with their families. Female gender and pre-fracture ability to walk by oneself tended to reduce the risk of mortality, but the effect was not statistically significant.

Many types of comorbidities have been reported to play a major role as predictive factors of mortality, including in-hospital, 30-day, and 1-year mortality, in patients with a hip fracture [3,16,18,22,23,24,25,27,37,38,45]. In our cohort, dementia or Alzheimer’s disease, chronic obstructive pulmonary disorder, and active malignancy were the strongest prognostic factors for mortality of Thai patients with a hip fracture. Chronic obstructive pulmonary disorder and active malignancy had previously been documented as prognostic factors in recent reports [3,25,27,37,45] as were dementia or Alzheimer’s [46,47,48]. Dementia or Alzheimer’s disease might disrupt cognitive function, reducing the return of self-care and walking ability and leading to an increase in mortality [47,49]. Underlying active malignancies included in the present study are lung, prostate, bladder, rectal, colon, hypopharynx, lip, and ovarian cancers. Although high ASA scores and the total number of comorbidities have been described as potent prognostic factors in previous reports [3,16,17], we considered that those factors might be redundant with the type of comorbidities. ASA scores in our cohort were determined by anesthesiologists only during the preoperative evaluation, so ASA scores were not available for patients who had conservative treatment. We decided to exclude ASA scores from the multivariable analysis because it appeared that diversity of disease severity and types of comorbidities would be a more meaningful measure.

In prior publications, laboratory evaluation results, including admission hemoglobin concentration, blood level of albumin, and Neutrophil to lymphocyte ratio (NLR), were found to be significantly related to 30-day or 1-year mortality in patients with a hip fracture [3,16,17,18,26,29]. Our results showed that an admission hemoglobin concentration of <10 g/dL was a significant predictor of mortality, a finding in concordance with those studies. Chronic inflammatory diseases, nonhematopoietic neoplasms, endocrinologic and metabolic disorders, blood loss, increased consumption or destruction of erythrocytes, lack of nutrients, and drug-induced anemia have been described as causes of low hemoglobin concentration in the elderly [50]. This study did not focus on identifying the causes of and specific treatments for patients who had a low hemoglobin concentration; however, our study did find that patients who had admission GFR < 30 mL/min/1.73 m^2^ had a mortality risk 3.5 times greater than those who had a higher GFR level. Although many studies have demonstrated that renal failure is one of the factors associated with in-hospital, 30-day, and 1-year mortality [18,22,23,25], we are of the opinion that admission GFR level might provide more insight into the severity of the disease than the term “renal failure or chronic kidney disease”. The cause of low GFR level might be acute or chronic renal failure [51,52]. A prerenal cause could be hemorrhage or volume depletion in acute renal failure; a postrenal cause could be bladder-outlet or ureteral obstruction. These possible causes of low hemoglobin concentration and low GFR level are potentially treatable and could reduce the mortality rate. For that reason, we considered hemoglobin concentration and GFR level to be modifiable factors.

Albumin level is one of the factors which has been found to be associated with mortality [17,29]. Unfortunately, the admission serum albumin level in patients was not routinely investigated at our institution during the time of this study: only 151 patients (19%) were examined for serum albumin level. For that reason, we decided not to include admission serum albumin level in the multivariable analysis.

A recent study reported that an admission NLR value >4.7, the marker used to evaluate systemic inflammation, is associated with higher 1-year mortality in elderly patients with a hip fracture [26], a result contrary to our study which found that an NLR value >4.7 was not significantly associated with mortality of Thai hip-fracture patients. In the prior study, NLR value was investigated in a specific population (50 female patients age between 65 and 80 years, ASA score 3, with an unstable intertrochanteric fracture treated with hemiarthroplasty and with a time between fracture and surgery of <72 h). Differences in the populations and other confounding factors might be responsible for the different results in the two studies.

Previous studies have reported that patients who received nonoperative treatment had a higher mortality rate compared to those who received surgical treatment [34,35,36,53]. Our study supports that finding. In the present study, Thai patients who had conservative treatment had a 3.5 times higher risk of mortality compared to patients receiving operative procedures in the multivariable survival model. Shorter time to surgery has previously been demonstrated to be a protective factor in reducing the mortality rate in many publications [18,20,22,35,37]. In the present study, however, a time from injury to operation ≥48 h showed an increasing trend in mortality in univariable analysis without statistical significance.

Nonoperative treatment, admission GFR < 30 mL/min/1.73 m^2^, admission hemoglobin concentration <10 g/dL, chronic obstructive pulmonary disorder, dementia or Alzheimer’s disease, and active malignancy were significant prognostic factors for higher all-cause mortality rates in Thai patients after a hip fracture with a C-statistic of 0.78. A recent study proposed a similar predictive model for in-hospital mortality following hip fractures in the elderly, but that study included advanced age, gender, congestive heart failure, asthma, rheumatologic disease, lung cancer, and antiaggregant medication as prognostic factors [24]. Evaluation of the predictive ability of that model provided a c-statistic of 0.77; however, limitations described in the study included a small sample size (38 patients with in-hospital mortality) which might have led to overfitting of the model. Another study reporting a prediction index for 1-year mortality after hip fracture included aged 90 and older, male sex, congestive heart failure, difficulty preparing meals, and not being able to drive as the predictors with a c-statistic of 0.73 [38]. In terms of discrimination power of the predictors, the proposed predictors in the current study had a relatively high predictive ability.

This study should be interpreted considering its strengths and limitations. On the strengths side, we intensively reviewed the patient medical charts, providing a level of detail equivalent to that of previous retrospective studies. Second, the independent prognostic factors included in this study demonstrated high predictive ability.

There are also some limitations in this study. First, the generalizability of the findings is restricted as the study focused exclusively on a Thai population. External validity will need to be evaluated in further studies to determine the applicability to other populations. Second, there were some missing patient data in this cohort, so we applied multiple imputation for variables which had more than 5% missing data. Third, the cause of death could not be ascertained for some patients in this study because mortality status was collected only from the date of death as recorded by the Thailand Civil Registration Office.

## 5. Conclusions

Types of comorbidities and laboratory evaluation results associated with mortality in Thai patients with a hip fracture include chronic obstructive pulmonary disorder, dementia or Alzheimer’s disease, active malignancy, admission GFR < 30 mL/min/1.73 m^2^, and admission hemoglobin concentration <10 g/dL. Thai hip-fracture patients who had these comorbidities or laboratory evaluation findings had risks of mortality 2.5, 4, 7, 3.5, and 2.5 times higher, respectively, than patients who did not. These factors could be used to develop a prognostic tool for Thai patients with hip fracture to help identify individuals with a higher risk of mortality.

## Figures and Tables

**Table 1:** Baseline clinical characteristics and potential predictors for all-cause mortality in Thai patients with fragility fracture of hip.

Characteristics	Dead *n*, (%) (*n =* 108)	Alive *n*, (%) (*n =* 667)	*p*-Value
**General Factors**			
Gender, *n* (%)			
Male	36 (33.33)	178 (26.69)	0.164
Female	72 (66.67)	489 (73.31)	
Age (years), Mean ± SD ^c^	81.63 ± 8.52	78.68 ± 9.65	0.003
Age at admission ≥85 years, *n* (%)	46 (42.59)	188 (28.19)	0.003
BMI ^a^ at admission (kg/m^2^), Mean ± SD ^c^ (*n =* 769)	19.82 ± 3.13	21.18 ± 4.05	<0.001
BMI ^a^ at admission ≥25 kg/m^2^, *n* (%) (*n =* 769)	8 (7.48)	96 (14.50)	0.048
Pre-fracture walking ability by oneself, *n* (%) (*n =* 606)	72 (91.14)	504 (95.64)	0.095
Living with family, *n* (%) (*n =* 774)	108 (100)	663 (99.55)	1.000
**Comorbidities**			
Active malignancy, *n* (%)	12 (11.11)	9 (1.35)	<0.001
Dementia or Alzheimer’s disease, *n* (%)	13 (12.04)	31 (4.65)	0.005
Hemiplegia, *n* (%)	2 (1.85)	34 (5.10)	0.213
Hypertension, *n* (%)	69 (63.89)	448 (67.17)	0.510
History of myocardial infraction, *n* (%)	4 (3.70)	11 (1.65)	0.144
Congestive heart failure, *n* (%)	2 (1.85)	5 (0.75)	0.254
Atrial fibrillation, *n* (%)	9 (8.33)	32 (4.80)	0.160
Chronic obstructive pulmonary disorder, *n* (%)	18 (16.67)	49 (7.35)	0.003
Diabetes mellitus, *n* (%)	36 (33.33)	185 (27.74)	0.251
Asthma, *n* (%)	1 (0.93)	16 (2.40)	0.492
Rheumatologic disease, *n* (%)	15 (13.89)	73 (10.94)	0.413
Cerebrovascular disease, *n* (%)	14 (12.96)	79 (11.34)	0.750
Current pneumonia, *n* (%)	1 (0.93)	5 (0.75)	0.595
Peptic ulcer, *n* (%)	3 (2.78)	9 (1.35)	0.227
ASA score ^b^, *n* (%) (*n =* 688)			<0.001
Class 1	0 (0)	7 (1.15)	
Class 2	29 (36.71)	397 (65.19)	
Class 3	49 (62.03)	202 (33.17)	
Class 4	1 (1.27)	3 (0.49)	
Type of fracture, *n* (%)			
Fractured neck of femur	32 (29.63)	255 (38.23)	0.107
Intertrochanteric fracture	76 (70.37)	412 (61.77)	
**Investigative Factors**			
Admission hemoglobin (g/dL), Mean ± SD ^c^	9.93 ± 1.89	10.85 ± 1.83	<0.001
Admission hemoglobin concentration <10 g/dL, *n* (%)	57 (52.78)	202 (30.28)	<0.001
Admission glomerular filtration rate (mL/min/1.73 m^2^), Mean ± SD ^c^	48.38 ± 56.90	67.31 ± 41.77	<0.001
Admission glomerular filtration rate <30 mL/min/1.73 m^2^, *n* (%)	36 (33.33)	87 (13.04)	<0.001
Admission serum albumin level (g/dL), Mean ± SD ^c^ (*n =* 151)	3.30 ± 0.63	3.58 ± 0.51	0.018
Neutrophil to lymphocyte ratio, Median ± IQR ^d^	7.36 ± 5.74	6.08 ± 5.90	0.276
Neutrophil to lymphocyte ratio ≥4.7, *n* (%)	73 (67.59)	433 (64.92)	0.663
**Treatment Factors**, *n* (%)			<0.001
Nonoperative treatment	29 (26.85)	58 (8.70)	
Dynamic hip screw	15 (13.89)	100 (14.99)	
Cephalomedullary nailing	36 (33.33)	262 (39.28)	
Stable angle plating	2 (1.85)	15 (2.25)	
Multiple screw fixation	2 (1.85)	38 (5.70)	
Arthroplasty	24 (22.22)	194 (29.09)	
Time from injury to operation (days), Median ± IQR ^d^ (*n =* 688)	9 ± 10	8 ± 8	0.020
Time from injury to operation ≥48 h, *n* (%) (*n =* 688)	78 (98.73)	586 (96.22)	0.508
Peripheral nerve or spinal block, *n* (%) (*n =* 688)	20 (25.32)	146 (23.97)	0.781

^a^ BMI = Body mass index, ^b^ ASA score = American Society of Anesthesiologists (ASA) Physical Status Classification, ^c^ SD = Standard deviation, ^d^ IQR = interquartile range.

**Table 2:** Univariable survival analysis of the potential predictors for all-cause mortality in Thai patients with fragility fracture of hip.

Characteristics	Hazard Ratio	95% Confidence Interval	*p*-Value
**General Factors**			
Male	1.36	0.91–2.03	0.131
Age at admission ≥85 years	1.83	1.25–2.69	0.002
BMI ^a^ at admission ≥25 kg/m^2^	0.50	0.24–1.02	0.056
Pre-fracture walking ability by oneself	0.52	0.24–1.13	0.101
**Comorbidities**			
Active malignancy	6.82	3.73–12.47	<0.001
Dementia or Alzheimer ’s disease	2.63	1.47–4.70	0.001
Hemiplegia	0.35	0.09–1.41	0.139
Hypertension	0.88	0.59–1.30	0.520
History of myocardial infraction	2.02	0.74–5.48	0.168
Congestive heart failure	2.34	0.57–9.47	0.234
Atrial fibrillation	1.82	0.92–3.60	0.086
Chronic obstructive pulmonary disorder	2.26	1.36–3.75	0.002
Diabetes mellitus	1.29	0.86–1.92	0.216
Asthma	0.44	0.06–3.18	0.419
Rheumatologic disease	1.31	0.76–2.25	0.336
Cerebrovascular disease	1.05	0.60–1.85	0.854
Current pneumonia	1.34	0.19–9.60	0.771
Peptic ulcer	2.26	0.72–7.12	0.164
ASA score ^b^ > 2	3.27	2.07–5.17	<0.001
Fracture neck of femur	0.67	0.45–1.01	0.062
**Investigative Factors**			
Admission hemoglobin concentration <10 g/dL	2.38	1.63–3.47	<0.001
Admission glomerular filtration rate <30 mL/min/1.73 m^2^	3.10	2.07–4.62	<0.001
Admission serum albumin level <3.5 g/dL	1.49	0.68–3.27	0.318
Neutrophil to lymphocyte ratio ≥4.7	1.14	0.76–1.70	0.534
**Treatment Factors**			
Nonoperative treatment	3.53	2.30–5.40	<0.001
Time from injury to operation ≥48 h	2.48	0.35–17.85	0.366
Peripheral nerve or spinal block	1.09	0.66–1.80	0.742

^a^ BMI = Body mass index, ^b^ ASA score = American Society of Anesthesiologists (ASA) Physical Status Classification.

**Table 3:** Multivariable analysis of the predictors for all-cause mortality in Thai patients with fragility fracture of hip (*N =* 775).

Characteristics	Hazard Ratio	95% Confidence Interval	*p*-Value
Nonoperative treatment	3.29	2.13–5.08	<0.001
Admission glomerular filtration rate <30 mL/min/1.73 m^2^	3.40	2.21–5.21	<0.001
Admission hemoglobin concentration <10 g/dL	2.31	1.57–3.40	<0.001
Chronic obstructive pulmonary disorder	2.63	1.56–4.42	<0.001
Dementia or Alzheimer’s disease	4.06	2.22–7.41	<0.001
Active malignancy	6.80	3.68–12.59	<0.001

C-statistic = 0.78.

## Supplementary Materials

The following are available online at https://www.mdpi.com/1010-660X/56/6/311/s1, Supplemental Material: The definitions of potential prognostic factors.
