# Supplementary material for: Prognostic Factors for All-Cause Mortality in Thai Patients with Fragility Fracture of Hip: Comorbidities and Laboratory Evaluations
Source: Medicina (Kaunas). 2020 Jun 24;56(6):311. doi: 10.3390/medicina56060311 (PMC7353872; doi:10.3390/medicina56060311)
Supplement: Supplementary file 1 [file medicina-56-00311-s001.pdf]

Supplemental Material: The definitions of potential prognostic factors

- General factors
  - Age at admission (continuous variable)
  - Age at admission ≥85 years [1] (binary variable)
  - Gender (male or female) (binary variable)
  - Body mass index (BMI) at admission (continuous variable)
  - BMI at admission ≥25 kg/meter^2 (the cut-off for obesity in the peoples of the Asia-Oceania region) [2] (binary variable)
  - Living with family (binary variable)
  - Pre-fracture walking ability by oneself defined as a patient who could walk independently either with or without gait aids (binary variable)
- Types of comorbidities [1,3-12]
  - Active malignancy, dementia or Alzheimer's disease, hemiplegia, hypertension, history of myocardial infarction, congestive heart failure, atrial fibrillation, chronic obstructive pulmonary disorder, asthma, current pneumonia, diabetes mellitus, rheumatologic disease, peptic ulcer (binary variables)
  - American Society of Anesthesiologists (ASA) Physical Status Classification (ASA score) which were interpreted by anesthesiologists (categorical variable)
  - The types of comorbidities in this study were determined based on the ICD-10 coding in the discharge summary.
- Type of fracture
  - Fracture neck of femur or intertrochanteric fracture (binary variable)
- Laboratory investigation results:
  - Admission hemoglobin concentration (continuous variable)
  - Admission hemoglobin concentration <10 g/dL. [3,5] (binary variable)
  - Admission *glomerular filtration rate (GFR)* (continuous variable)
  - Admission *glomerular filtration rate <30* ml/min/1.73m^2 (binary variable)
  - *A*dmission serum albumin level (continuous variable)
  - Admission neutrophil to lymphocyte ratio (NLR) (continuous variable)
  - Neutrophil to lymphocyte ratio ≥4.7 [13] (binary variable)
- Treatment factors
  - Type of treatment: non-operative treatment, dynamic hip screw, cephalomedullary nailing, stable angle plating, multiple screw fixation, arthroplasty (categorical variable)
  - Time from injury to operation defined as the number of days from the date of the injury to the date of the operation (continuous variable)
  - Time from injury to operation ≥48 hours [4,10] (binary variable)
  - Use of spinal or nerve block (binary variable)

References

1. Karres, J.; Kieviet, N.; Eerenberg, J.P.; Vrouenraets, B.C. Predicting Early Mortality After Hip Fracture Surgery: The Hip Fracture Estimator of Mortality Amsterdam. *J Orthop Trauma* **2018**, *32*, 27-33, doi:10.1097/BOT.0000000000001025.
2. Kanazawa, M.; Yoshiike, N.; Osaka, T.; Numba, Y.; Zimmet, P.; Inoue, S. Criteria and classification of obesity in Japan and Asia-Oceania. *World Rev Nutr Diet* **2005**, *94*, 1-12, doi:10.1159/000088200.
3. Maxwell, M.J.; Moran, C.G.; Moppett, I.K. Development and validation of a preoperative scoring system to predict 30 day mortality in patients undergoing hip fracture surgery. *Br J Anaesth* **2008**, *101*, 511-517, doi:10.1093/bja/aen236.

4. Khan, M.A.; Hossain, F.S.; Ahmed, I.; Muthukumar, N.; Mohsen, A. Predictors of early mortality after hip fracture surgery. *Int Orthop* **2013**, *37*, 2119-2124, doi:10.1007/s00264-013-2068-1.
5. Nijmeijer, W.S.; Folbert, E.C.; Vermeer, M.; Slaets, J.P.; Hegeman, J.H. Prediction of early mortality following hip fracture surgery in frail elderly: The Almelo Hip Fracture Score (AHFS). *Injury* **2016**, *47*, 2138-2143, doi:10.1016/j.injury.2016.07.022.
6. Endo, A.; Baer, H.J.; Nagao, M.; Weaver, M.J. Prediction Model of In-Hospital Mortality After Hip Fracture Surgery. *J Orthop Trauma* **2018**, *32*, 34-38, doi:10.1097/BOT.0000000000001026.
7. Lo, L.W.T.; Yanling, X.; Chou, A.C.C.; Howe, T.S.; Allen, J.C.; Koh, J.S.B. End-Stage Renal Failure Is an Independent Risk Factor for 1-Year Mortality After Hip Fracture Surgery. *Geriatr Orthop Surg Rehabil* **2018**, *9*, 2151459318770561, doi:10.1177/2151459318770561.
8. Sanz-Reig, J.; Salvador Marin, J.; Ferrandez Martinez, J.; Orozco Beltran, D.; Martinez Lopez, J.F.; Quesada Rico, J.A. Prognostic factors and predictive model for in-hospital mortality following hip fractures in the elderly. *Chin J Traumatol* **2018**, *21*, 163-169, doi:10.1016/j.cjtee.2017.10.006.
9. Traven, S.A.; Reeves, R.A.; Althoff, A.D.; Slone, H.S.; Walton, Z.J. New 5-Factor Modified Frailty Index Predicts Morbidity and Mortality in Geriatric Hip Fractures. *J Orthop Trauma* **2019**, 10.1097/BOT.0000000000001455, doi:10.1097/BOT.0000000000001455.
10. Chang, W.; Lv, H.; Feng, C.; Yuwen, P.; Wei, N.; Chen, W.; Zhang, Y. Preventable risk factors of mortality after hip fracture surgery: Systematic review and meta-analysis. *Int J Surg* **2018**, *52*, 320-328, doi:10.1016/j.ijsu.2018.02.061.
11. Cenzer, I.S.; Tang, V.; Boscardin, W.J.; Smith, A.K.; Ritchie, C.; Wallhagen, M.I.; Espaldon, R.; Covinsky, K.E. One-Year Mortality After Hip Fracture: Development and Validation of a Prognostic Index. *J Am Geriatr Soc* **2016**, *64*, 1863-1868, doi:10.1111/jgs.14237.
12. Harstedt, M.; Rogmark, C.; Sutton, R.; Melander, O.; Fedorowski, A. Impact of comorbidity on 6-month hospital readmission and mortality after hip fracture surgery. *Injury* **2015**, *46*, 713-718, doi:10.1016/j.injury.2014.12.024.
13. Temiz, A.; Ersozlu, S. Admission neutrophil-to-lymphocyte ratio and postoperative mortality in elderly patients with hip fracture. *Ulus Travma Acil Cerrahi Derg* **2019**, *25*, 71-74, doi:10.5505/tjtes.2018.94572.
